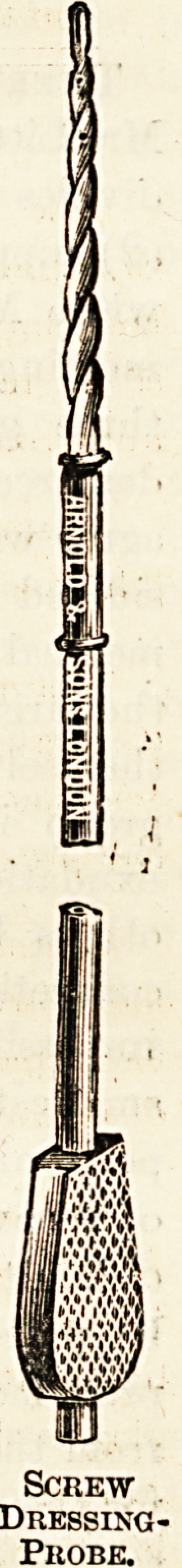# Chronic Cervical Catarrh of the Uterus

**Published:** 1900-11-10

**Authors:** 


### Hospital Clinics and Medical Progress.

### CHRONIC CERVICAL CATARRH OF THE UTERUS.—*Continued.*

Combined with the systematic douching already de scribed the application of tampons to the cervix is often desirable. While prolonged hot douching promotes circulatory changes in the pelvis by dilating the vessels which subsequently become contracted by reflex action, tamponnage may be used to deplete the local circulation. Glycerine withdraws fluid from the vessels in the immediate neighbourhood of its application, and conveys drugs conveniently to the parts affected. So we find in erosions, or catarrhal patches, of the cervix a tampon of absorbent cotton soaked in glycerine of borax, or covered with glycerole of lead, or a 10 per cent. solution of ichthyol in glycerine, promotes rapid covering of the erosion with normal epithelium. A piece of cotton may be tied with silk, tape, or string into the size of a bantam's egg, and dipped in one of the preparations just mentioned. It may be passed into the vagina through a Sims or Fergusson speculum, or that unnecessary contrivance, a tampon introducer, and pushed up till it lies against the cervix. As a matter of practice it is perfectly easy to do this without any instrument whatever. Place the patient in the Sims position, hold the tampon with your right hand, and introduce one or two fingers of the left hand into the vagina. Pull back the posterior vaginal wall very steadily, and you dilate the vagina sufficiently, and painlessly, to enable you to pass the tampon well up to the cervix. Leave the end of the attached string hanging an inch or so outside the vulva, and tell the woman to remove the tampon by pulling on this string in 12 to 24 hours. It is a little matter, but it is as well to advise her to wear a sanitary towel while the tampon is in place, for there may be a free discharge from the vagina as a result of the insertion of the plug or tampon. You may insert these plugs once a week or once a day, according to the condition of the parts, but two or three in the week are usually found sufficient. Further treatment is pursued by means of the speculum, and for general use Sims' speculum is by far the most convenient to insert and to work with. It is not so handy as a Fergusson for applying local baths and lotions to the uterus or for some dressings; but taken all round, where you have few opportunities of familiarising yourself with surgical appliances, it is better to limit one's armamentarium, and the Sims is universally useful.

Put the patient, then, on her left side near the edge of the couch, her head on a cushion and her left arm beneath and behind her, over the edge of the couch, and her knees drawn up. Throw a light shawl or rug over her, and let your attendant draw up the patient's clothing and expose the vulva sufficiently, but not unnecessarily. Now take the speculum in your right hand, dip the blade you wish to insert into 1-40 carbolic lotion to warm it, or warm and smear with some convenient antiseptic lubricant; separate the labia with the fingers of the left hand, and press the speculum against the posterior part of the orifice of the vagina. It readily enters and slips upwards and backwards to the vaginal vault. By tilting the upper end forward you will probably press the cervix within view, when it will be easy to observe and treat it. You will find it a convenience to steady it by a uterine hook or volsellum caught in one or other lip, by means of which you can also evert the lip sufficiently to observe the cervical canal more or less. If the cervix be lacerated a hook or volsellum inserted in either lip (the attendant holding the speculum in the desired position) will enable you to roll its lips outwards and note the extent of the laceration, and inwards again to place the cervix in its normal position, and understand what is required to restore this permanently.

Having noted the erosions and the lacerations of the cervix, you will inspect the cervical canal. This will probably be found more or less filled with the white glairy mucus already described. Roll a thin piece of absorbent cotton on an aseptic game skewer, or on one of my uterine screw dressing probes, which are much more easily covered and cleaned than Playfair's, and twist it round within the cervix until the mucus be removed. Now you will observe the roughened surface, and probably some of the ovula Nabothii, or little retention cysts. These are best punctured with a scarifier or with the point of a clean bistoury and the contents evacuated. After again cleaning the canal with wool, dry or moistened with 1 in 20 carbolic lotion, a little pure carbolic acid or iodized phenol may be applied to it thoroughly on a probe or skewer wrapped round with cotton. To do this effectually with a Sims speculum, fix the uterus with a hook or volsellum in one lip, place a little absorbent cotton under the cervix to catch any excess of the caustic, and dip the end of your covered probe in the dressing fluid selected, which is now used to thoroughly swab the cervical canal. Any excess is absorbed by the dry portion of wool on the probe and the wad you have placed beneath the cervix, and the vagina is not damaged by it. Now mop the cervix with more dry cotton and apply a tampon soaked in glycerine, or 15 per cent. ichthyol ammon. in glycerine, and the dressing is complete. This is to be repeated every week or fortnight, or in bad cases oftener even. If the cervix be large and thickened, a few punctures in it made by a scarifier or bistoury to the depth of $\frac{1}{8}$ inch and followed by a warm douche will help to deplete it, and give a good deal of relief.

Screw Dressing-Probe.

Sometimes in nulliparæ the os is short and pointed, and the cervix bulged and distended with retained secretion. In these cases it may be impossible to obtain access to the cervical canal for the application of dressings or the removal of mucus until the cervix is divided bilaterally with scissors. Take care that the vagina has been thoroughly disinfected with douches of 1-2000 mercuric chloride or iodide previously, and that these douches are kept up once or twice a day until healing is well advanced, or you may add cellulitis to the other troubles; and keep the incision well below the vaginal vault, or free bleeding may occur.

In milder cases it is not necessary to use strong caustics to the cervical canal. Chloride of zinc, 20 or 40 grains to the ounce of distilled water, does very well; or nitrate of silver solution of the same strength. The essentials of any form of treatment adopted are patient perseverance in it, and attention to local asepsis and to judicious consti-

B

tutional remedies. But some cases are extremely obstinate and call for severer measures. You will scarcely cure cervical catarrh, for example, if lacerations are not previously repaired, and the cicatricial tissue in their angles removed.

Very chronic cases call for curettage, at least, and frequently the affected glands cannot be destroyed by the ordinary caustics, and must be excised. So with a much hypertrophied cervix that has existed for some time and grows very little smaller under patient treatment. Here amputation is the only satisfactory treatment. But we will discuss these more radical methods in another paper.

## Figures and Tables

**Figure f1:**